# Prognosis prediction and risk stratification of breast cancer patients based on a mitochondria-related gene signature

**DOI:** 10.1038/s41598-024-52981-w

**Published:** 2024-02-03

**Authors:** Yang Wang, Ding-yuan Wang, Ke-na Bu, Ji-dong Gao, Bai-lin Zhang

**Affiliations:** 1 Department of Breast Surgery, National Cancer Center/National Clinical Research Center for Cancer/Cancer Hospital, Chinese Academy of Medical Sciences and Peking Union Medical College, Beijing, 100021 China; 2 Xingyuan Hospital of Yulin City, Yulin City, 719051 Shanxi Province China; 3 Department of Breast Surgery, National Cancer Center/National Clinical Research Center for Cancer/Cancer Hospital & Shenzhen Hospital, Chinese Academy of Medical Sciences and Peking Union College, Shenzhen, 518116 China

**Keywords:** Cancer, Breast cancer, Cell death and immune response, Tumour immunology, Computational biology and bioinformatics, Data processing, Data publication and archiving, Databases, Probabilistic data networks

## Abstract

As the malignancy with the highest global incidence, breast cancer represents a significant threat to women’s health. Recent advances have shed light on the importance of mitochondrial function in cancer, particularly in metabolic reprogramming within tumors. Recognizing this, we developed a novel risk signature based on mitochondrial-related genes to improve prognosis prediction and risk stratification in breast cancer patients. In this study, transcriptome data and clinical features of breast cancer samples were extracted from two sources: the TCGA, serving as the training set, and the METABRIC, used as the independent validation set. We developed the signature using LASSO-Cox regression and assessed its prognostic efficacy via ROC curves. Furthermore, the signature was integrated with clinical features to create a Nomogram model, whose accuracy was validated through clinical calibration curves and decision curve analysis. To further elucidate prognostic variations between high and low-risk groups, we conducted functional enrichment and immune infiltration analyses. Additionally, the study encompassed a comparison of mutation landscapes and drug sensitivity, providing a comprehensive understanding of the differing characteristics in these groups. Conclusively, we established a risk signature comprising 8 mitochondrial-related genes—ACSL1, ALDH2, MTHFD2, MRPL13, TP53AIP1, SLC1A1, ME3, and BCL2A1. This signature was identified as an independent risk predictor for breast cancer patient survival, exhibiting a significant high hazard ratio (HR = 3.028, 95%CI 2.038–4.499, *P* < 0.001). Patients in the low-risk group showed a more favorable prognosis, with enhanced immune infiltration, distinct mutation landscapes, and greater sensitivity to anti-tumor drugs. In contrast, the high-risk group exhibited an adverse trend in these aspects. This risk signature represents a novel and effective prognostic indicator, suggesting valuable insights for patient stratification in breast cancer.

## Introduction

According to GLOBOCAN 2020, female breast cancer has now overtaken lung cancer as the most prevalent malignant tumor globally, with an estimated 2.26 million new cases annually^1^. This alarming statistic highlights the urgent need for effective treatment strategies. Recent advancements in imaging screening^2^, surgical techniques^3^, radiotherapy^4^, and therapeutic drugs have markedly improved the prognosis for breast cancer patients. Nonetheless, the complexity and heterogeneity of breast cancer necessitate personalized treatment approaches^5^. The development of microarray and next-generation sequencing technologies has enabled polygenic testing, providing clinicians with additional clinical insights^6^. The significance of polygenic testing in breast cancer management has grown considerably, ranging from PAM50 for molecular typing^7^, to the 21-gene (Oncotype DX Breast Recurrence Score®)^8^ and MammaPrint™ 70-gene signature^9^, both of which are crucial in assessing clinical risk and informing treatment decisions in breast cancer cases.

Mitochondrial dynamics and signaling significantly influence cellular metabolism and are pivotal in numerous diseases^10^, including metabolic disorders like diabetes and obesity^11^. As central players in the metabolism of fatty acids, amino acids, and nucleotides, mitochondria have been found to be crucial in the development and progression of cancer^12^. The substantial influence of mitochondrial metabolism on various stages of tumorigenesis, including malignant transformation, tumor progression, and therapeutic response, is well-established^13,14^. Consequently, numerous strategies have been developed to target abnormalities in mitochondrial metabolism between cancerous and normal cells^15,16^. For instance, one study revealed that targeting mitochondrial metabolism in breast cancer can increase the sensitivity of these and potentially other tumor types to mitochondrial inhibitors^17^. In the context of triple-negative breast cancer (TNBC), which has limited treatment options, targeting mitochondrial metabolism has also emerged as a promising therapeutic approach^18,19^. Moreover, research has shown the anti-tumor potential of mitochondrial transplantation in breast cancer cells, which is achieved through precise regulation of mitochondrial function^20^. In addition to these therapeutic applications, mitochondria play a pivotal role in the metabolism and activation of both immune and cancer cells^21^. Furthermore, the role of mitochondrial acquisition and increased oxidative phosphorylation in cancer progression and chemotherapy resistance has also been identified^22^.

In this study, we utilized a comprehensive multi-scale bioinformatics approach to develop a breast cancer risk signature based on mitochondrial-related genes. The clinical application of this signature was validated through various methods, including Kaplan–Meier analysis, Nomograms, DCA Curves, and Calibration Curves, enabling the effective stratification of breast cancer patients into high-risk and low-risk groups. To further understand the underlying mechanisms, we conducted a comprehensive analysis of genes differentially expressed between these risk groups, using Gene Ontology (GO), Kyoto Encyclopedia of Genes and Genomes (KEGG) pathways, and Gene Set Enrichment Analysis (GSEA) to identify involved biological pathways. Additionally, we investigated the correlation between immune infiltration and our risk signature. In summary, our research provides novel insights into mitochondrial related genes in breast cancer, which hold potential for the development of prognostic biomarkers for diagnosis and treatment.

## Materials and methods

### Application of TCGA and METABRIC and collection of mitochondria-related genes

The transcriptomic and corresponding survival data of breast cancer patients from the TCGA samples were downloaded from the UCSC Xena database (https://xenabrowser.net/datapages/). We selected the TCGA database for its comprehensive genomic data and high-quality, standardized information on breast cancer. We also acquired mutation data and clinical characteristics through the “TCGAbiolinks” package. In our study, male patients were excluded due to the distinct clinical and molecular characteristics of breast cancer in males, which differ significantly from the female cases. Additionally, cases with missing clinical and survival data were omitted to ensure the integrity and accuracy of our analysis. After matching the TCGA barcodes, the remaining cases was divided into two groups: primary tumor (1036 cases) and adjacent-normal tissues (99 cases).

To identify mitochondrial-related genes (MRGs), we utilized the Molecular Signature Database v7.5 (MSigDB) (http://www.gsea-msigdb.org/gsea/msigdb), from which we acquired a dataset (M9577) comprising 450 genes^23^. Within the TCGA database, we matched these 450 genes and yielding 418 MRGs for differential expression analysis.

For external validation, RNA-transcriptomic data of breast cancer were downloaded from the METABRIC database (https://www.cbioportal.org/datasets). The METABRIC database was specifically chosen for its extensive clinical data and long-term patient follow-up, providing an ideal resource for external validation of our findings. It is important to note that the METABRIC database served solely for external validation in our analysis and was not used for other processes like differential analysis or model fitting. We have detailed relevant grouping information and clinicopathological characteristics in Table 1.

**Table 1:** Clinicopathological characteristics of the BC cases in TCGA and MERABRIC datasets.

Characteristic	TCGA (n = 1036)	METABRIC (n = 1903)	
Age (mean (SD))	58.05 (12.93)		61.09 (12.98)
Status (%)			
Alive	888 (85.7)		801 (42.1)
Dead	148 (14.3)		1102 (57.9)
PT (%)		Tumor size (%)	
T1	269 (26.0)	≤ 2 cm	820 (43.5)
T2 T2	600 7.9)	> 2 cm & ≤ 5 cm	968 (51.4)
T3	127 (12.3)	> 5 cm	95 (5.0)
T4	37 (3.6)		
Tx	3 (0.3)		
PN (%)		Positive nodes (%)	
N0	485 (46.8)	0 nodes	992 (52.1)
N1	345 (33.3)	1–3 nodes	604 (31.7)
N2	117 (11.3)	4–9 nodes	204 (10.7)
N3	72 (6.9)	≥ 10 nodes	103 (5.4)
Nx	17 (1.6)		
PM (%)			
M0	861 (83.1)		
M1	27 (2.6)		
Mx	148 (14.3)		
Stage (%)		Stage (%)	
Stage I	173 (16.8)	Stage I	474 (33.8)
Stage II	589 (57.1)	Stage II	800 (57.1)
Stage III	238 (23.1)	Stage III	115 (8.2)
Stage IV	19 (1.8)	Stage IV	9 (0.6)
Stage X	12 (1.2)	Stage 0	4 (0.3)
Subtype (%)			
BRCA_LumA	484 (46.7)	BRCA_LumA	678 (35.6)
BRCA_LumB	187 (18.1)	BRCA_LumB	461 (24.2)
BRCA_Her2	72 (6.9)	BRCA_Her2	220 (11.6)
BRCA_Basal	165 (15.9)	BRCA_Basal	199 (10.5)
Unknown	128 (12.4)	Unknown	146 (7.7)
		Claudin-low	199 (10.5)

The TCGA cohort was used as training cohort and MERABRIC cohort as the validation cohort. PT, pathological tumor stage; PN, pathological node stage; PM, pathological metastasis stage.

### Construction and clinical application of the risk signature

Differential gene analysis was conducted between tumor and adjacent-normal tissues using the “DESeq2” package, following criteria of |Log2Fold Change|> 1 and adjusted-*P* < 0.05. As a result, 64 differentially expressed mitochondrial-related genes (DE-MRGs) were identified, including 39 upregulated and 25 downregulated genes. Their prognostic significance in breast cancer was determined through univariate Cox regression analysis, leading to the identification of 9 genes with significant prognostic implications. The approach was then refined by the application of LASSO regression, a method known for its efficiency in reducing complexity and enhancing precision. Ultimately, a risk signature consisting of 8 genes was established. The risk formula was as follows:

$$ risk \;score\;\left( {written \;as \;riskScore} \right) = \mathop \sum \limits_{i = 1}^{n} Coef\left( i \right)*Expr\left( i \right) $$

Based on the median riskScore, patients were stratified into high and low risk groups. Survival analysis was performed using Kaplan–Meier (K–M) curves and the log-rank test. The predictive power of risk signature was assessed by Receiver Operating Characteristic (ROC) analysis, time-dependent ROC, and area under the curve (AUC) evaluations. The association between risk groups and clinical characteristic was demonstrated using the “ggpubr” package. Moreover, a Nomogram model was constructed based on Cox regression analyses. The model’s discriminative ability was evaluated by calculating the concordance index (C-index), and its performance was further validated through Decision Curve Analysis (DCA) and Calibration Curves.

### Differential gene and functional enrichment analyses between risk groups

Differential expression analysis was performed to identify genes that showed significant differential expression between high and low risk groups. Functional enrichment analysis of these genes was then conducted to explore variations in biological functions across the risk groups. This included Gene Ontology (GO) analysis^24^, which focused on Biological Processes (BP), Molecular Functions (MF), and Cellular Components (CC). Additionally, Kyoto Encyclopedia of Genes and Genomes (KEGG) pathway analysis^25^ was utilized to identify specific pathways significantly affected in different risk groups. Complementing these, Gene Set Enrichment Analysis (GSEA) ^23,26^ was applied to detect predefined gene function terms that exhibited notable differences between the risk groups. These analyses were facilitated using the “clusterProfiler^27^”, “msigdbr” and “fgsea” packages.

### Comprehensive analysis of tumor immune microenvironment

The Estimation of Stromal and Immune cells in Malignant Tumor tissues (ESTIMATE) ^28^ algorithm (https://sourceforge.net/projects/estimateproject) is designed to deduce tumor cell content and distinguish infiltrating normal cells by leveraging unique transcriptional profiles of cancer samples. In our analysis, this algorithm was utilized to assess the expression levels of stromal and immune signatures, which facilitating to calculation of patient-specific scores. Additionally, CIBERSORT^29^ tool (https://rdrr.io/github/singha53/amritr/src/R/supportFunc_cibersort.R) was employed to estimate the composition of 22 distinct types of tumor-infiltrating immune cells (TIICs) in each tissue sample. Consequently, a correlation analysis was conducted between these cells and the riskScore, with the results presented in scatter plots and a significance threshold set at *P* < 0.05. Furthermore, TCGA breast cancer samples were classified into different immune subtypes based on immune-related gene expression^30^. This comparison was visualized using column graphs and evaluated using a Chi-square test, processed with the “ImmuneSubtypeClassifier” package. Additionally, single-sample GSEA (ssGSEA) ^31^ was performed to define enrichment scores, and the abundance of 28 tumor-infiltrating immune cells (TIICs) in each sample was evaluated based on a gene set of 782 genes.

### Mutation landscape and weighted correlation network analysis (WGCNA)

After matching with TCGA barcodes, the top 15 most frequently mutated genes in different risk groups were visualized using oncoplots. We then delved deeper by examining somatic interactions, and extended our analysis to include survival analysis and the calculation of tumor mutational burden (TMB) specific to each risk group. These analyses and visualizations were processed using the “maftools” package. Furthermore, to identify the genes most significantly associated with immune infiltration and riskScore levels, we employed the “WGCNA” package, which allowed for effective screening of relevant module genes.

### Treatment response and drug sensitivity

The impact of treatment on prognosis across different risk groups was illustrated using K–M curves with the Log-rank test. Drug sensitivity training data were downloaded from Genomics of Drug Sensitivity in Cancer (GDSC, https://www.cancerrxgene.org) ^32^. These data were then validated with TCGA data, leading to the identification of commonly used antitumor drugs for our analysis. The results of this analysis were effectively presented through radar plots, utilizing the “oncoPredict” and “ggpubr” packages.

### Statistical analysis

All statistical analyses and visualizations in this study were performed using R software (version 4.2.0). Adobe Illustrator was employed for image combination. Student’s *t* test was used for statistical comparisons. The Wilcoxon test was applied to detect differences in gene expression and immune infiltration scores. Variations in riskScore across different clinical features were assessed using the Kruskal‒Wallis’s test. K–M curves in the survival analysis were evaluated with the log-rank test. Additionally, correlation plots were generated using Spearman’s correlation test. *P* < 0.05 was considered statistically significant.

### Ethical declarations

The current study investigated publicly available data, and no ethical approval was needed. All methods were carried out in accordance with the Declaration of Helsinki.

### Consent to participate

The current study investigated publicly available data, and no consent to participate was needed.

## Results

### Preliminary screening of prognostic mitochondrial genes

**Figure 1:**
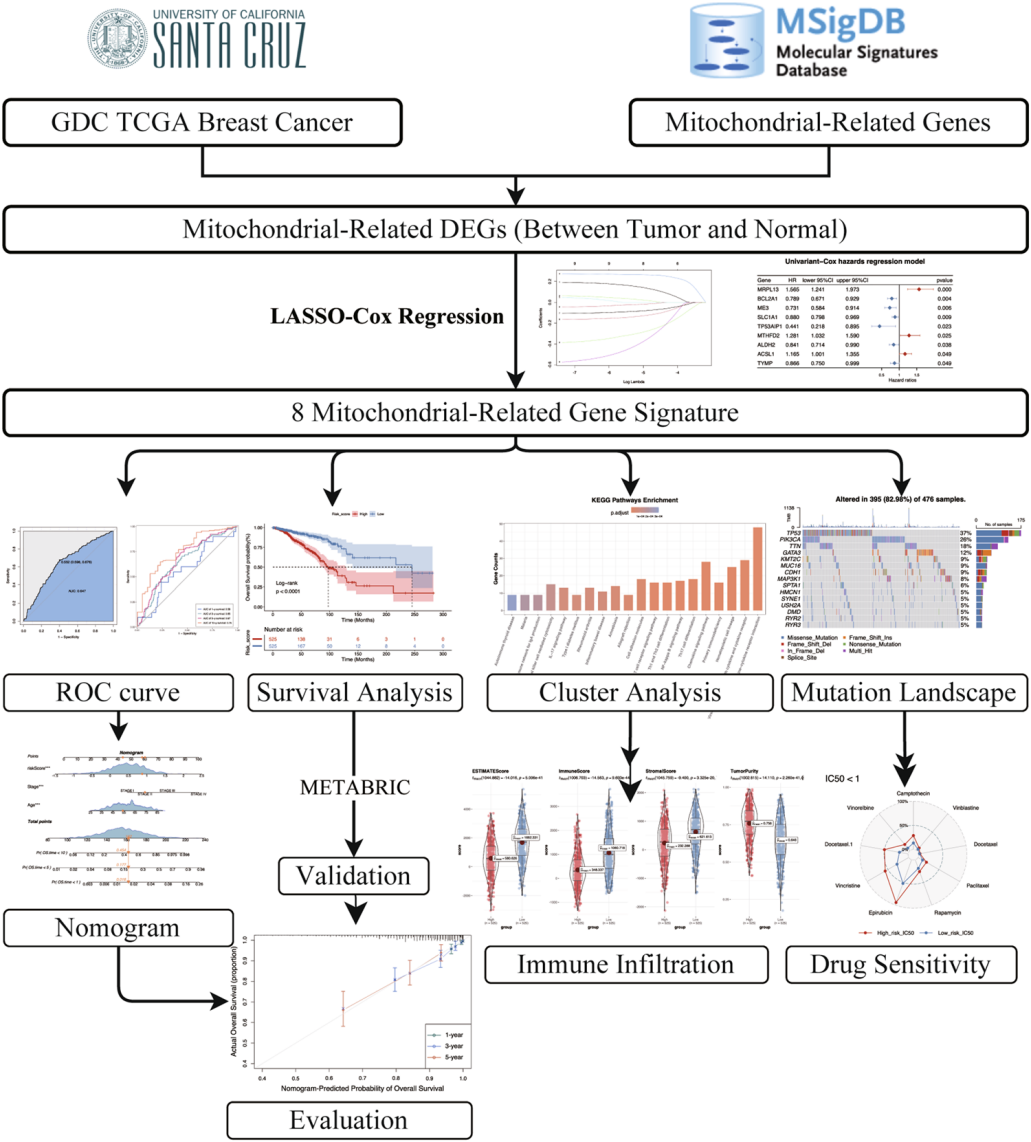
The overall analysis workflow of this study. Schematic flowchart of the workflow performed to build and validate the breast cancer prognostic risk signature. Some typical analysis results were shown in reduced size (normal-sized pictures are shown later).

**Figure 2:**
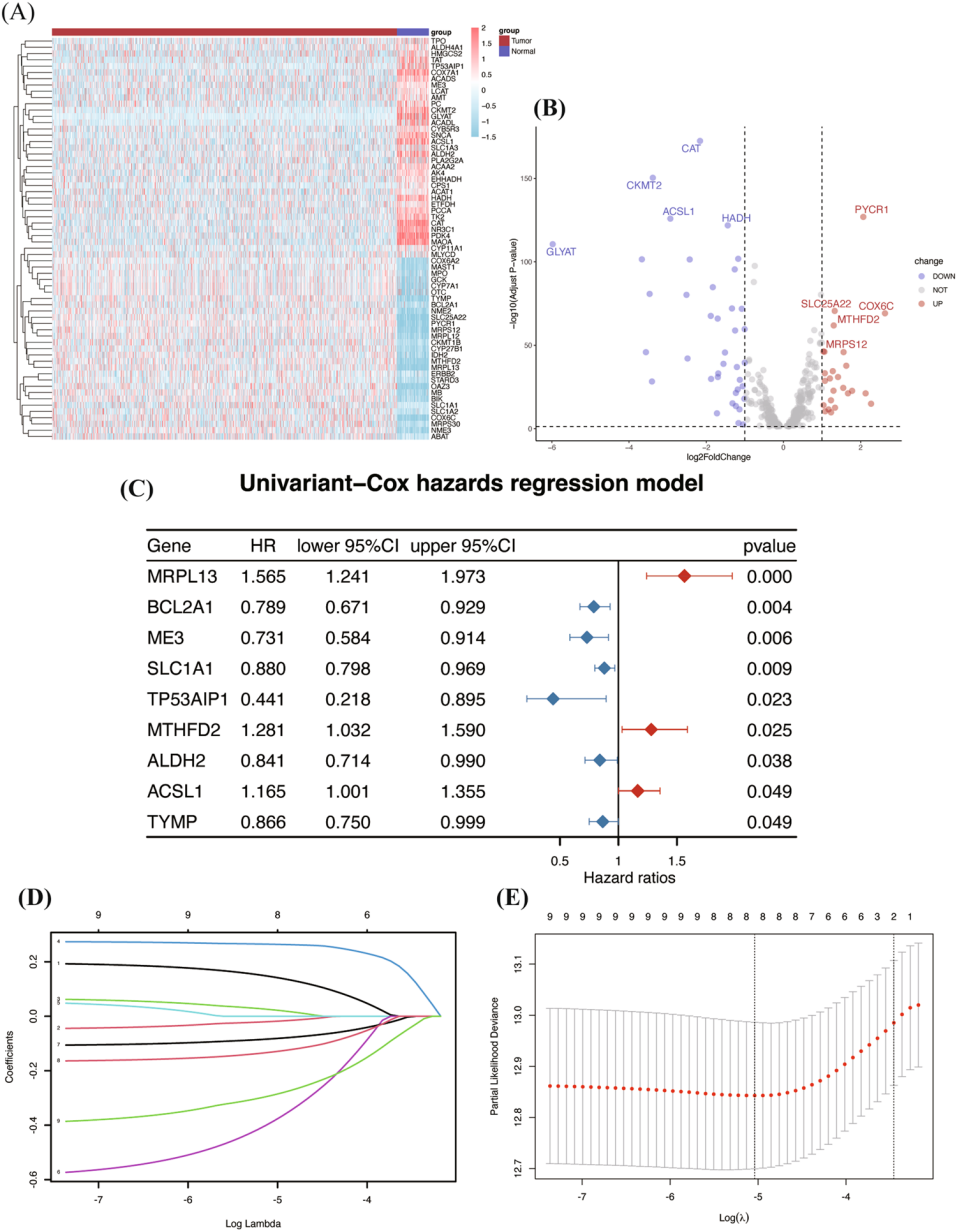
Differential distribution of MRGs and profile plot of LASSO regression. (**A**) Differential distribution of MRGs between tumor and adjacent-normal tissues in the TCGA cohort. This heatmap was created by R software (version 4.2.0) and the “pheatmap” package. (**B**) Volcano plot of the DE-MRGs. (**C**) Univariate Cox regression identified MRGs significantly related to OS (*P* < 0.05). (**D**) LASSO coefficient profile plots of the 8-MRG risk signature. (**E**) Penalty plot for the LASSO regression for the MRGs with error bars denoting the standard errors. MRG, mitochondria-related genes; DE-MRGs, differentially expressed MRGs; LASSO, least absolute shrinkage and selection operator.

The analytical process of this study is depicted in Fig. 1. We analyzed 418 mitochondrial-related genes (MRGs) for differential expression, identifying 64 differentially expressed MRGs (DE-MRGs). The expression patterns of these DE-MRGs are illustrated in a heatmap (Fig. 2A). Subsequently, we prioritized these DE-MRGs based on their *P* values, selecting the top 5 upregulated and downregulated genes for presentation in a volcano plot (Fig. 2B). Further, we subjected these 64 DE-MRGs to univariate Cox regression analysis in breast cancer patients, revealing 9 genes (MRPL13, BCL2A1, ME3, SLC1A1, TP53AIP1, MTHFD2, ALDH2, ACSL1, TYMP) significantly associated with overall survival (OS), as demonstrated in a forest plot (Fig. 2C).

### Construction of the risk gene signature by LASSO

In our study, LASSO regression was chosen to construct the risk signature. This method is particularly effective in handling high-dimensional data, crucial for genomic studies. It allows for efficient variable selection, reducing overfitting and enhancing the robustness of the model^33^. Applying this technique, we successfully isolated the most predictive prognostic genes from an extensive dataset. In particular, our LASSO regression analysis was specifically focused on 9 DE-MRGs (Fig. 2D–E), leading to the identification of 8 genes that constitute the MRG risk signature.

We calculated the riskScore using the following formula: riskScore = (0.153 × expr_ACSL1) + (0.022 × expr_ALDH2) + (0.029 × expr_MTHFD2) + (0.265 × expr_MRPL13) + (− 0.417 × expr_TP53AIP1) + (− 0.086 × expr_SLC1A1) + (− 0.137 × expr_ME3) + (− 0.302 × expr_BCL2A1). (The detailed descriptions of these 8 MRGs^34–50^ are provided in Table 2).

**Table 2:** Brief description of 8 MRGs.

Gene	Full name	Description
ACSL1	Acyl-CoA Synthetase Long-Chain Family Member 1	Exists at the outer mitochondrial membrane and plays an important role in ferroptosis in diverse cancer cell types (Ellis et al. ^36^; Wang et al. ^37^; Coleman^35^; Beatty et al. ^34^; Wang et al. ^37^)
ALDH2	Aldehyde Dehydrogenase 2	Aldh2-deficient can activate a variety of carcinogenic pathways and promote the occurrence of hepatocellular carcinoma (Seo et al. ^39^)
MTHFD2	Methylenetetrahydrofolate dehydrogenase 2	Regulates purine synthesis and signal transduction in activated T cells to promote proliferation and induces immune invasion of cancer cells by upregulating PD-L1 (Huang et al. ^40^ ; Shang et al. ^41^; Sugiura et al. ^42^)
MRPL13	Mitochondrial ribosomal protein L13	Exists in the mitochondria of eukaryotic cells, a poor prognostic factor for BC (Tao et al. ^44^; Cai et al. ^43^; Ye and Zhang ^45^)
TP53AIP1	Tumor Protein P53 Regulated Apoptosis Inducing Protein 1	TP53 target, plays a key role in inducing apoptosis in response to UV-induced DNA damage (Benfodda et al. ^46^)
SLC1A1	Solute Carrier Family 1 Member 1	An extracellular glutamine transporter, promotes tumor growth through reprogramming glutamine metabolism of natural killer T-cell lymphoma, while rendering tumor cells sensitive to asparaginase treatment (Xiong et al. ^47^)
ME3	Malic Enzyme 3	Catalyzes oxidative decarboxylation of (S)-malate to pyruvate and reverse the decarboxylation reaction. Involved in the carcinogenesis of pancreatic cancer (Zhang et al. ^48^)
BCL2A1	BCL2 Related Protein A1	Bcl-2 family member, regulates endogenous apoptosis and target anti-apoptotic members (Li et al. ^49^; Murthy et al. ^50^)

### Evaluation and expression pattern of the risk signature in TCGA cohort

**Figure 3:**
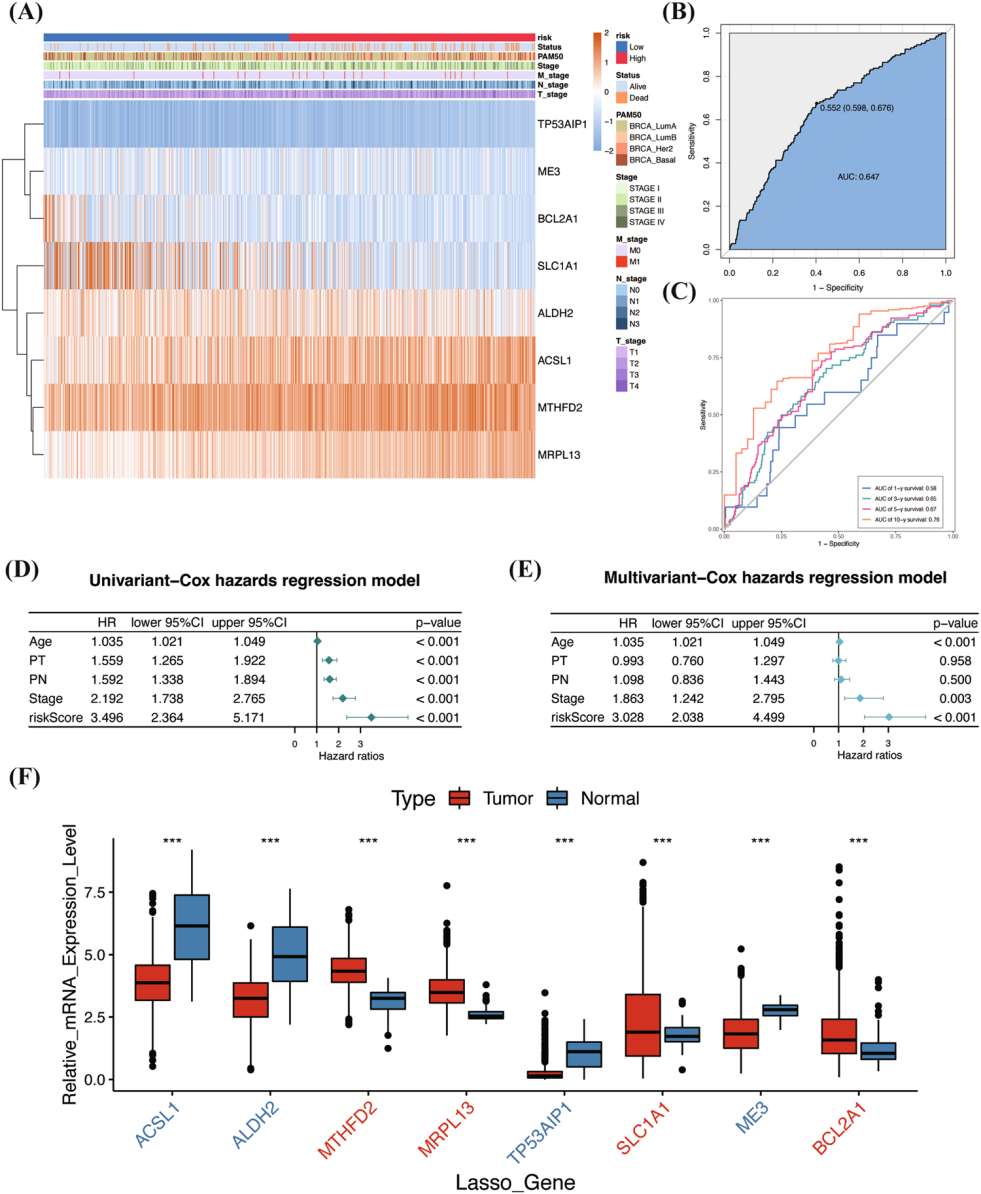
MRG risk signature and clinical application. (**A**) 8 MRGs and distribution heatmap of clinical features. This heatmap was created by R software (version 4.2.0) and the “pheatmap” package. (**B**, **C**) ROC and time-ROC analysis of the risk signature. (**D**, **E**) Univariate and multivariate Cox regression for the riskScore and corresponding clinical features. (**F**) 8 MRG expression levels between tumor and adjacent-normal tissues. (The plot annotations were as follows: * if *P* < 0.05, ** if *P* < 0.01, and *** if *P* < 0.001 and ns if nonsignificant.).

A heatmap effectively displayed the correlation between the 8 genes and clinical characteristics, with the riskScore arranged from the lowest to highest (Fig. 3A). The overall Area Under the Curve (AUC) was 0.647 (Fig. 3B). Notably, the model demonstrated an increasing predictive power for survival over time, reflected in the ascending AUC values at 1, 3, 5, and 10 years, which were 0.58, 0.65, 0.67, and 0.76, respectively (Fig. 3C). In the subsequent univariate Cox regression analysis, variables such as Age, Pathological Tumor stage (PT), Pathological Node stage (PN), AJCC stage, and riskScore were involved. The hazard ratios (HRs) and *P* values are presented in a forest map (Fig. 3D), highlighting their significant prognostic impacts. Remarkably, the riskScore was identified as the most significant and effective prognostic factor (HR = 3.496, 95%CI:2.364–5.171, *P* < 0.001), surpassing others like Age (HR = 1.035, 95%CI:1.021–1.049, *P* < 0.001), PT (HR = 1.559, 95%CI:1.265–1.922, *P* < 0.001), PN (HR = 1.592, 95%CI:1.338–1.894, *P* < 0.001) and AJCC stage (HR = 2.192, 95%CI:1.738–2.765, *P* < 0.001). In the further multivariate Cox regression analysis, which adjusted for cofactors, it was established that Age (HR = 1.035, 95% CI: 1.021–1.049, *P* < 0.001), AJCC stage (HR = 1.863, 95% CI: 1.242–2.795, *P* = 0.003), and riskScore (HR = 3.028, 95% CI: 2.038–4.499, *P* < 0.001) as independent survival predictors (Fig. 3E).

Concerning the 8 MRGs expression pattern, 4 genes, namely ACSL1, ALDH2, TP53AIP1, and ME3, were observed to be downregulated in tumor tissues while being upregulated in adjacent-normal tissues. Conversely, the remaining four genes—MTHFD2, MRPL13, SLC1A1, and BCL2A1—showed opposite expression patterns (Fig. 3F). Additionally, a boxplot was employed to depict the expression levels of these MRGs across different risk groups, classified based on the median riskScore (Supplementary Fig. 1A).

### Clinical application of the risk signature

**Figure 4:**
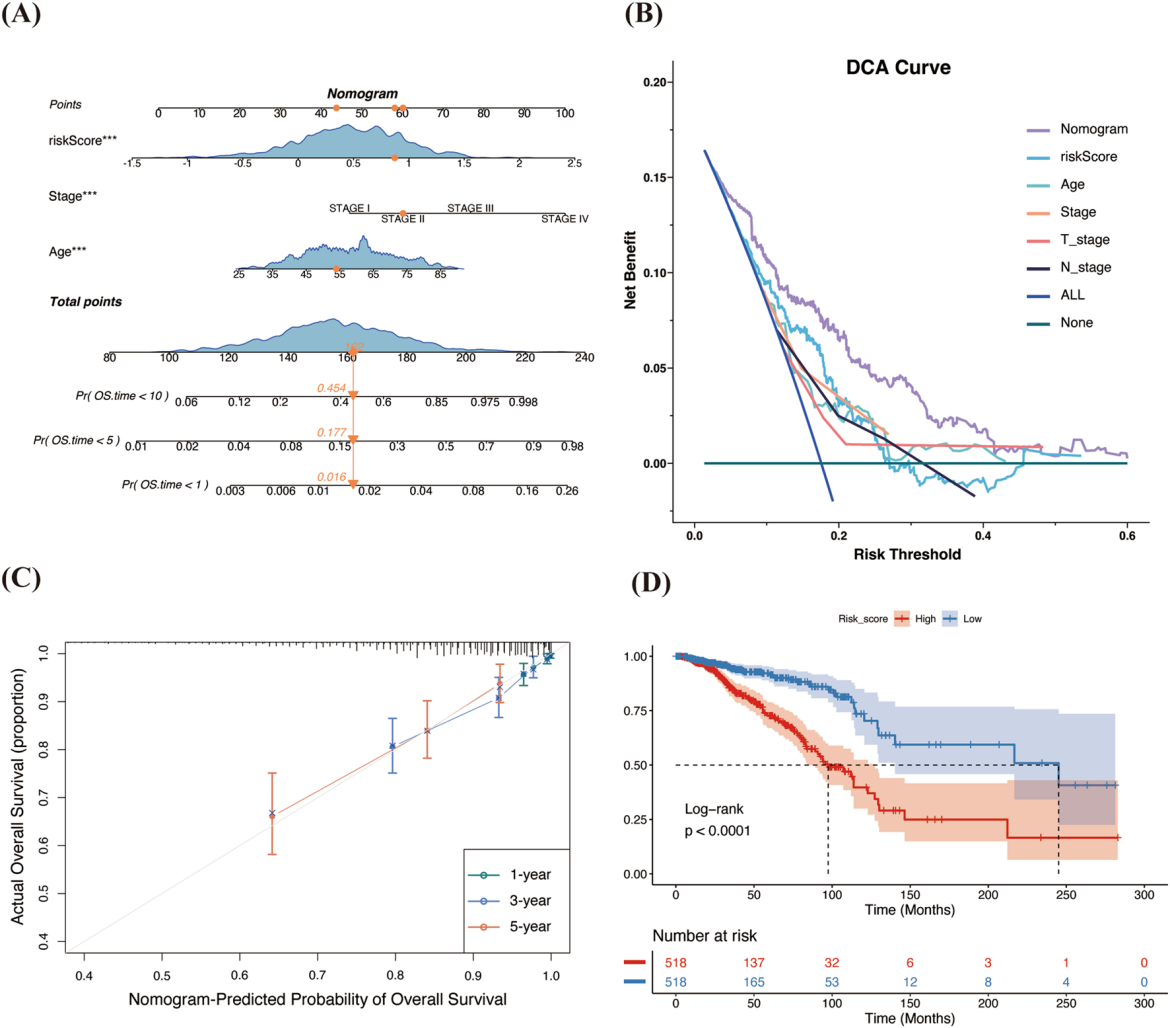
Construction and evaluation of the risk signature-based nomogram model. (**A**) Age, AJCC stage, and riskScore (independent risk factors) were enrolled in the Nomogram, and the total score could be used as a tool to predict the 1-, 5-, and 10-year prognosis of breast cancer patients. The orange dots and arrows in the plot represent the clinical characteristics of a patient and the survival probability of the corresponding year. (**B**) DCA of the Nomogram. (**C**) Calibration curve of the Nomogram to predict survival at 1 year, 3 years, and 5 years. (**D**) K–M curves for the prognosis analysis between high and low risk groups. DCA, decision curve analysis.

We developed a Nomogram model incorporating independent risk factors (Age, AJCC stage, riskScore) (Fig. 4A). This model provides clinicians with a quantitative method to predict patient outcomes more reliably. To assess the predictive performance of our Nomogram, we employed the concordance index (C-index), which is a vital statistical tool in the evaluation of predictive models, particularly in the context of survival analysis. Our Nomogram achieved a C-index of 0.763 (standard error 0.045), indicating robust predictive power. Furthermore, we performed the Decision Curve Analysis (DCA) to evaluate the clinical application of the Nomogram. The DCA curve demonstrated a higher net benefit across a range of threshold probabilities when compared to the use of single predictors alone (Fig. 4B). Lastly, the Calibration Curve was employed to compare the predicted survival with the observed outcomes at 1-year, 3-year, and 5-year intervals (Fig. 4C). This analysis revealed a high level of accuracy in the Nomogram’s ability to predict survival at these time points, underscoring its potential values in clinical settings.

### Comparative analysis of RiskScore correlation with clinical features in TCGA and METABRIC cohorts

Our study utilized the TCGA database exclusively as a training set, while the METABRIC database was employed for external validation. Initially, we evaluated patient survival in the TCGA cohort by K–M curves for different risk groups. We discovered that patients in the low-risk group demonstrated significantly better prognoses (Log-rank *P* < 0.001, Fig. 4D), with their survival status depicted in a scatter plot (Supplementary Fig. 1B). Further, we examined the relationship between riskScore and various clinical characteristics, including PT, PN, AJCC staging, and PAM50 subtypes. The Kruskal–Wallis’s test identified significant statistical differences among these factors in the TCGA cohort, suggesting an association between higher riskScore and more advanced disease features, such as T4, N3, Stage IV (Supplementary Fig. 1C–F).

In the external validation using the METABRIC cohort, the risk signature derived from TCGA was applied to calculate riskScore. The survival analysis for the METABRIC cohort echoed the TCGA results, showing improved outcomes for patients in the low-risk group (Supplementary Fig. 2A, B). Notably, tumor size and positive lymph node count corresponded to T and N stages in METABRIC cohort. Similar to TCGA, METABRIC patients with the higher riskScore often presented with advanced features, like having 10 positive lymph nodes. Notably, patients with the more aggressive molecular subtypes such as basal-like and Her2 positive demonstrated higher riskScore in METABRIC cohort, while this trend was less apparent in the TCGA cohort. However, in both cohorts, patients with the less aggressive Luminal A breast cancer presented the lowest riskScore (Supplementary Fig. 3A–D).

In terms of the prognostic effects of each gene within this signature, our analysis revealed that 3 genes reached statistical significance in influencing prognosis in TCGA cohorts. High expression of ME3 and TP53AIP1 was associated with a significantly better prognosis (ME3: Log-rank *P* = 0.013, TP53AIP1: Log-rank *P* < 0.0001), whereas high MRPL13 expression correlated with a worse prognosis (Log-rank *P* = 0.0017, Supplementary Fig. 4).

### Functional enrichment analyses between risk groups

**Figure 5:**
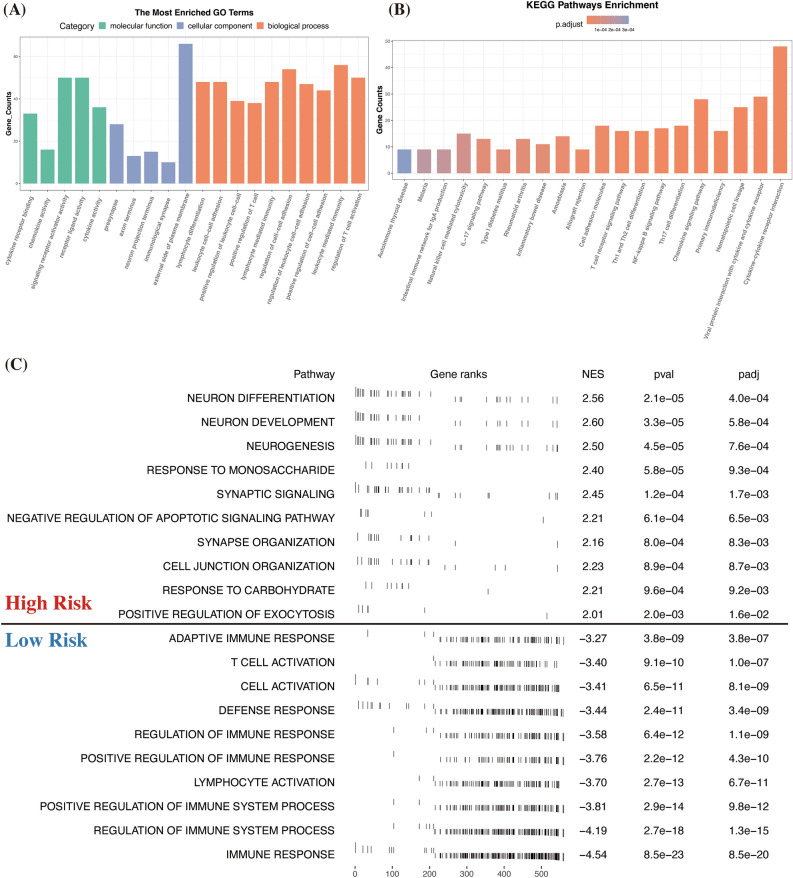
Functional enrichment analyses of the different risk groups. (**A–C**) GO, KEGG, and GSEA analyses of the DEGs between the high- and low-risk groups. GO, Gene Ontology; KEGG, Kyoto Encyclopedia of Genes and Genomes; GSEA, Gene set enrichment analysis.

Our risk signature effectively classifies patients into high-risk and low-risk groups, subsequently led us to investigate potential biological functional differences between these groups. We conducted differential gene expression analysis for both groups and then performed functional enrichment analyses on the identified genes. This approach aimed to elucidate the underlying factors contributing to the prognostic disparities observed in these patients. Through analysis using GO and KEGG pathways, we found that the genes differentially expressed between the groups were primarily associated with T-cell activation, leukocyte-mediated immunity, and the NF-kappa B signaling pathway (Fig. 5A, B). Furthermore, GSEA results indicated that low-risk patients showed enrichment in the positive regulation of immune response and immune system processes. In contrast, high-risk patients exhibited predominant features in neuronal differentiation and development (Fig. 5C).

### Assessment of the ability of the risk signature to distinguish different immune infiltrations

**Figure 6:**
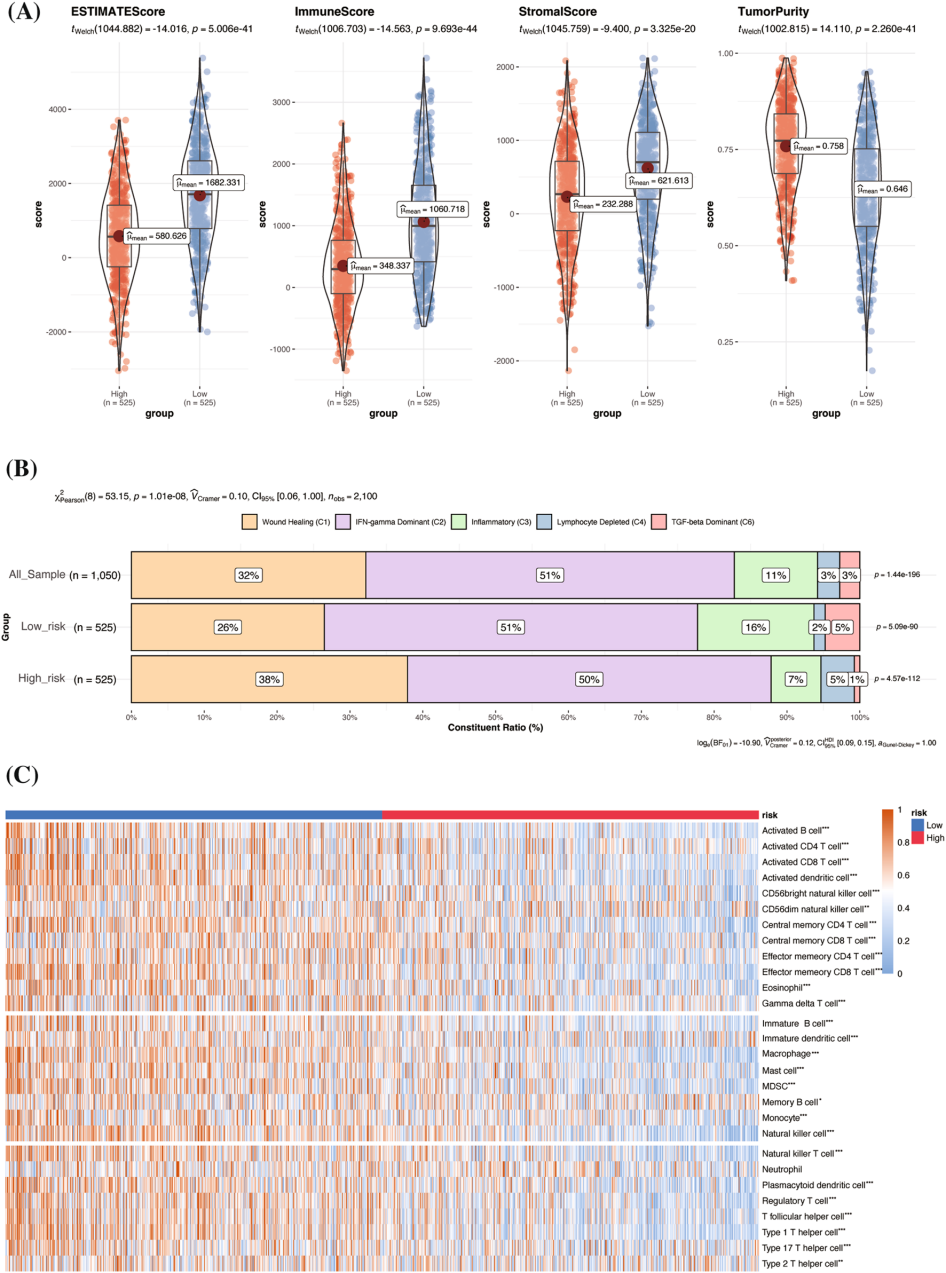
Immune infiltration of the risk groups. (**A**) The ESTIMATE algorithm presented the immune infiltration scores across risk groups, including the ESTIMATE score, immune score, stromal score and tumor purity. (**B**) Immune subtype of TCGA breast cancer cases based on immune-related gene expression and the different subtype proportions in risk groups. (**C**) ssGSEA demonstrated the abundance of 28 tumor-infiltrating immune cells in individual tissue samples by a heatmap ranked from the lowest to the highest riskScore. (This heatmap was created by R software (version 4.2.0) and the “pheatmap” package. The plot annotations were as follows: * if *P* < 0.05, ** if *P* < 0.01, and *** if *P* < 0.001 and ns if nonsignificant). ESTIMATE Estimation of Stromal and Immune cells in Malignant Tumor tissues using Expression.

The ESTIMATE analysis revealed that low-risk patients had significantly higher ESTIMATE scores, along with elevated immune and stromal scores, and lower tumor purity (Fig. 6A). Following this, we utilized CIBERSORT to analyze tumor-infiltrating immune cells, identifying those significantly correlated with riskScore from 22 types of immune cells (Supplementary Fig. 5A–L). Furthermore, we classified the breast cancer samples in the TCGA cohort into five distinct immune subtypes: C1 (Wound Healing, 32%), C2 (IFN-gamma Dominant 51%), C3 (Inflammatory, 11%), C4 (Lymphocyte Depleted, 3%), and C6 (TGF-beta Dominant, 3%). Notably, the low-risk group showed a lower proportion of C1 subtype but higher proportions of C3 and C6. In contrast, high-risk patients exhibited a higher proportion of C1 and lower proportions of C3 and C6 (Fig. 6B). Additionally, the ssGSEA results indicated significant differences in immune cell enrichment between the high-risk and low-risk groups, except for neutrophils (Fig. 6C).

### Risk signature and prognosis of different gene mutation landscape

**Figure 7:**
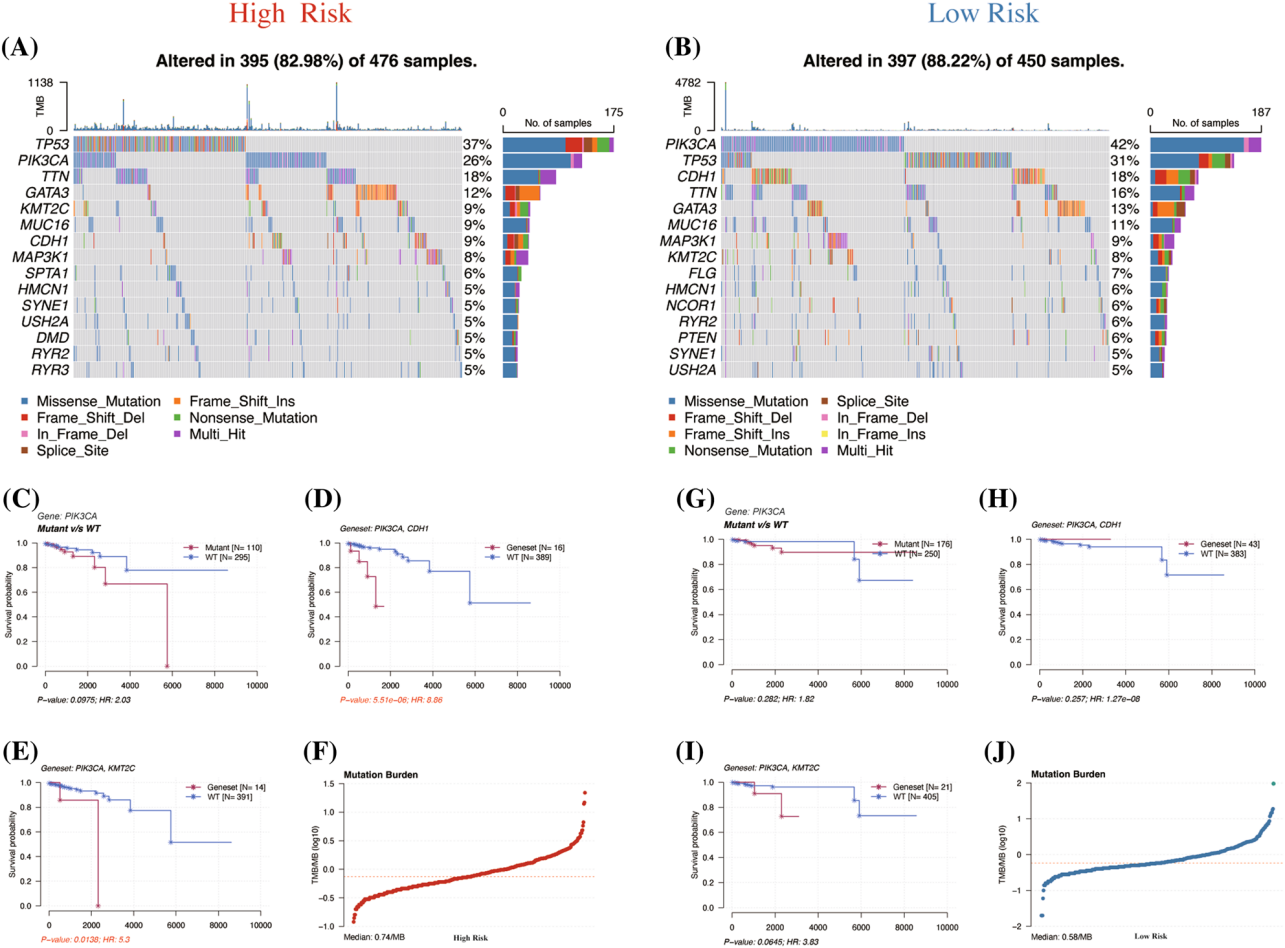
Mutation landscape analysis between risk signatures. (**A**, **B**) Oncoplots for the different mutation landscapes of risk groups. (**C**–**E** & **G**–**I**) K–M curve for the most frequently affected gene and combination of associated genes affecting prognosis in the high and low risk groups. (**F**, **J**) The calculated TMB in high and low risk groups. TMB, Tumor mutational burden.

Patients within the risk groups were matched using sample barcodes, allowing us to illustrate the mutation landscape through oncoplots (Fig. 7A, B). Focusing on PIK3CA and its associated genes impacting prognosis, CDH1 and KMT2C emerged as the top two genes, ranked by their *P* values. In our survival analysis, mutations in the PIK3CA gene (HR = 2.03, *P* = 0.0975) and its co-mutation with CDH1 (HR = 8.86, *P* < 0.001) and KMT2C (HR = 5.3, *P* = 0.0138) were linked to poorer prognosis in high-risk patients (Fig. 7C–E). However, in the low-risk group, these same genetic alterations did not demonstrate a significant worse prognosis in survival outcomes (Fig. 7G–I). Moreover, we calculated the tumor mutational burden (TMB), finding it to be higher in high-risk patients (0.74/MB) compared to low-risk patients (0.58/MB) (Fig. 7F, J).

### Weighted gene co-expression network analysis

**Figure 8:**
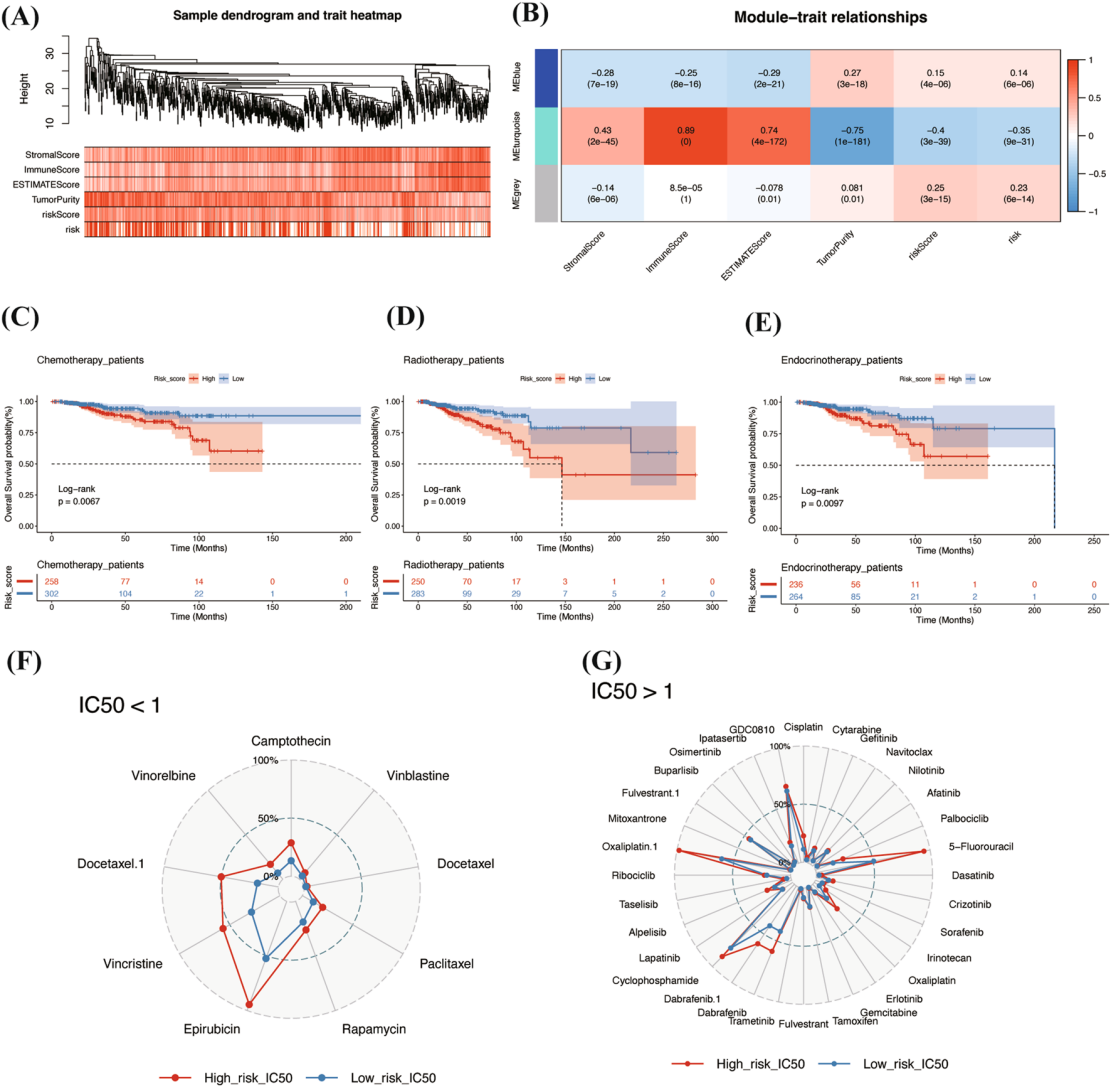
WGCNA, treatment responses and drug sensitivity. (**A**) Dendrogram trait heatmap for the overall situation between individual cases after WGCNA clustering and ESTIMATE, riskScore, and risk group. This heatmap was created by R software (version 4.2.0) and the “WGCNA” package. (**B**) Three color module genes clustered by WGCNA and corresponding statistical correlation. (**C**–**E**) K–M curves for the prognostic analysis of patients with chemotherapy, radiotherapy and endocrinotherapy among different risk groups in the TCGA cohort. (**F**–**G**) Radar plots for drug sensitivity between different risk groups among IC50 < 1 & IC50 > 1. WGCNA, weighted gene co-expression network analysis.

In our study, we employed Weighted Gene Co-expression Network Analysis (WGCNA) to identify co-expression modules correlated with clinical features in breast cancer. The clustering of individual cases, alongside ESTIMATE scores, riskScore, and risk group classifications, was visualized in a sample dendrogram trait heatmap (Fig. 8A). Among the identified modules, the turquoise module emerged as particularly significant. It exhibited a positive correlation with the ESTIMATE scores, encompassing both stromal and immune scores, indicating its potential relevance in the tumor microenvironment. Conversely, this module showed a negative correlation with tumor purity, higher riskScore, and categorization into the high-risk group (Fig. 8B). To further explore the functional implications of the turquoise module, we performed a comprehensive Gene Ontology (GO) analysis. This analysis revealed that variations in immune regulation could be attributed to the genes within the turquoise module (Supplementary Fig. 6A,B).

### Treatment response and drug sensitivity

In our study, we utilized data from the TCGA and METABRIC cohorts to evaluate treatment responses and drug sensitivity in breast cancer patients. Our analysis revealed that high-risk patients in both cohorts demonstrated a significantly worse prognosis in chemotherapy, radiotherapy, and endocrine therapy (Fig. 8C–E and Supplementary Fig. 6C–E). To further our understanding, we closely examined the sensitivity of various risk groups to commonly used clinical treatments. We found that patients classified as high-risk (the red line) consistently showed higher IC50 values in both IC50 > 1 and IC50 < 1 groups. This indicates a lower sensitivity to treatment, particularly to standard breast cancer chemotherapy drugs like Epirubicin, Docetaxel, and Paclitaxel (Fig. 8F–G).

## Discussion

In the past decade, the reprogramming of metabolism has garnered significant attention^51^, as evidenced by numerous studies highlighting altered mitochondrial metabolism as a key mechanism of therapeutic resistance in cancer treatment^52–55^. Meanwhile, the development of polygenic models targeting mitochondrial-related genes using bioinformatics methods is a promising direction in the diagnosis, treatment, and prevention of breast cancer. Recently, Luo et al.^56^ constructed a gene prediction model using the mitochondrial GOBP term gene set, combined with overall survival (OS) analysis. However, it is important to note that this study did not differentiate between tumor and adjacent-normal tissues and lacked an exploration of the link between these genes and clinical applications. Another study established a signature based on mitochondrial function-related long non-coding RNA (lncRNA), offering crucial insights for prognosis and targeted immunotherapy in breast cancer at non-coding RNA level^57^. Despite these significant contributions, they also highlight areas where further research is needed, particularly in transcriptomic and clinical translation.

Our methodology initiated with a differential analysis contrasting tumor tissue against adjacent-normal tissue. Subsequently, we incorporate this analysis with the LASSO-Cox algorithm to establish an 8 Mitochondrial-Related-Genes risk signature. Our mitochondrial gene-based risk model achieved an overall Area Under the Curve (AUC) of 0.647. A detailed examination of the AUC over time intervals of 1, 3, 5, and 10 years revealed an increasing trend, from 0.58 to 0.76 (Fig. 3B, C). This trend underscores the signature’s consistent accuracy in predicting long-term breast cancer prognosis. This performance surpasses the moderate accuracy of established models like BCRAT (Breast Cancer Risk Assessment Tool) and BCSC (Breast Cancer Surveillance Consortium), which have a maximum AUC of 0.71^58^. The integration of mitochondrial genetics into breast cancer risk assessment introduces a novel dimension, potentially enhancing clinical decision-making. The innovation of our approach stems from exploiting the complex relationship between mitochondrial dysfunction and tumorigenesis. This offers a unique perspective in cancer risk prediction and highlights our model’s cutting-edge contribution to the dynamic field of cancer biology.

Recognizing the signature’s effective predictive power, we constructed a Nomogram model by amalgamating the results of multivariate Cox regression analysis. Decision Curve Analysis (DCA) is an established method for evaluating nomograms, tailored to address the practical requirements of clinical decision-making^59^. Additionally, a Calibration Curve serves as another means to assess the alignment between predicted outcomes and actual observations. These methodologies collectively affirmed the efficacy of our nomogram model, which is rooted in the risk signature. Moreover, our findings indicate that high-risk patients experience markedly poorer prognoses, with a higher riskScore being positively associated with more aggressive clinical attributes. These results were further substantiated through verification in the METABRIC validation cohort (Supplementary Figs. 1–3).

Within the 8 Mitochondrial-Related Genes (MRGs) analyzed, ACSL1, MTHFD2, and MRPL13 were notably overexpressed in both the high-risk group and tumor tissues. Conversely, TP53AIP1 and ME3 exhibited higher expression in the low-risk group and adjacent-normal tissues. Specifically, a higher expression of ME3 and TP53AIP1 was associated with significantly better prognosis, whereas elevated MRPL13 levels correlated with worse outcomes. This suggests that TP53AIP1 and ME3 may function as tumor suppressors, and MRPL13 might act as an oncogene in breast cancer. Aligning with our findings, one study confirmed TP53AIP1 as a novel tumor suppressor gene in breast cancer, potentially offering a new therapeutic target^60^. Additionally, another study identified high MRPL13 expression as a poor prognostic factor in breast cancer, proposing its use both as a prognostic marker and a potential therapeutic target^43,44^.

To understand the prognostic differences between high and low-risk groups, we conducted a detailed analysis of differentially expressed genes (DEGs) between these groups, followed by functional enrichment analyses. In GO analysis, most BP terms were linked to immune responses. In addition, KEGG analysis revealed that Th17-cell differentiation and the NF-kappa B signaling pathway, along with most other terms, were intimately linked to immune responses and oncogenesis. Complementing these findings, GSEA highlighted the up-regulation of several immune-related elements in the low-risk group. This suggests that a lower riskScore, potentially influenced by the mitochondria’s involvement in immune response and cell death^45,61^, may be indicative of a more robust immune response. Conversely, in the high-risk group, terms associated with neuron differentiation were predominant. This aspect may be attributed to the role of nerve growth factor in the proliferation, invasion, and metastasis of breast cancer cells, especially in TNBC^62–64^.

Therefore, we further analyzed the relationship between immune infiltration and our risk signature. In our analysis, we initially applied the ESTIMATE algorithm to quantify the proportions of tumor cells, infiltrating immune cells, and stromal cells within the tumor immune microenvironment. In line with prior observations, patients classified as low-risk demonstrated elevated ESTIMATE scores, indicative of increased immune and stromal cell presence and reduced tumor purity. This suggests a more significant degree of immune infiltration in these patients, typifying a “warmer” tumor status. Furthermore, using CIBERSORT, we assessed the infiltration levels of 22 immune cell types (Supplementary Fig. 5). Notably, a significant negative correlation was observed between the riskScore and CD8 T cells (R = − 0.26, *P* < 2.2e−10), which play a vital role in tumor destruction^65^. In contrast, M2 macrophages, known for facilitating angiogenesis and neovascularization^66^, showed a significant positive correlation with the riskScore (R = 0.19, *P* = 2e−10). These M2 macrophages are implicated in stromal activation and remodeling^67^, contributing to tumor-promoting activities including immunosuppression^68^, thus potential correlating with poorer prognosis in breast cancer patients. Additionally, the riskScore was negatively correlated with M1 macrophages, recognized for their role in enhancing antitumor inflammatory responses and as key elements in proinflammatory reactions^69^.

In our extended immunotyping analysis, we observed that C1 subtype prevalence was higher in high-risk patients (38% vs. 26%), while C3 subtype was more prevalent in low-risk patients (16% vs 7%). The C1 subtype, characterized as “Wound Healing immune”, exhibits high expression of angiogenic genes and Th2 cells. This subtype is associated with high tumor cell proliferation and significant intratumoral heterogeneity, often leading to less favorable outcomes. Conversely, the C3 subtype, known as “inflammatory”, is defined by elevated Th17 and Th1 gene expressions. It typically shows low to moderate tumor cell proliferation and minimal intratumoral heterogeneity, correlating with the best prognosis among the subtypes. Aligning with these findings, a low riskScore correlates with increased immune cell infiltration, indicative of a “warmer” immune microenvironment in low-risk patients. Hence, it is plausible to infer that our risk signature can distinguish patients with varying immune responses, and this diverse tumor immune status may explain the differences in prognosis.

We also investigated the mutational landscape differences between the risk groups. Notably, TP53 mutations were predominantly observed in high-risk patients (37%), whereas PIK3CA mutations were more common in low-risk patients (42%). The TP53 mutation is linked with high mutation frequencies in TNBC and HER2-positive subtypes, at 80% and 70% respectively, and considerably lower in luminal A and B types, at 10% and 30% respectively^7,70–72^. Conversely, the PIK3CA mutation, recognized as a genomic marker of breast cancer^73^, exhibits lower mutation rates in TNBC (16%) compared to HR + /HER2- (42%) and HER2 + (31%) breast cancer^74^. These patterns partially elucidate the variations in riskScore across molecular subtypes. Furthermore, the same gene mutation status is associated with a significantly worse prognosis in high-risk patients, potentially linked to their generally poorer prognosis. The increased TMB observed in high-risk patients may be attributable to the higher prevalence of TP53 mutations and the greater proportion of TNBC^75^. We employed WGCNA to elucidate genes linked to prognostic variations in breast cancer and discovered that the gene expression trends in the turquoise module closely mirrored our hypotheses. Further GO analysis of these genes indicated that differences in immune regulation might be a contributing factor (Supplementary Fig. 6A, B).

In our final examination focusing on the therapeutic aspects of breast cancer, we observed significant prognostic disparities between different risk groups. Specifically, patients classified in the high-risk cohort exhibited poorer outcomes across three types of treatments, a trend consistent in both the TCGA and METABRIC cohorts. Building on these findings, we further analyzed drug sensitivity, revealing that the low-risk group showed greater sensitivity to a range of chemotherapy drugs commonly used in breast cancer treatment, such as paclitaxel, docetaxel, and Epirubicin. This enhanced responsiveness to chemotherapy in the low-risk group could help explain the variations in treatment outcomes, illustrating the utility of our gene signature in guiding therapeutic decisions.

However, there are some limitations to our study. Firstly, while our risk signature is valuable for assessing patient survival and identifying individuals at high risk of breast cancer, its utility in early diagnosis should be approached with caution. Additionally, considering the hormone-dependent characteristics of breast cancer, it is crucial to investigate the applicability of our findings to other hormone-related female cancers, such as ovarian and cervical cancers, in future research. Furthermore, the predominance of white individuals (70%) in the large databases used for our analysis underscores a limitation in racial diversity, emphasizing the need for developing localized data sets to more comprehensively validate our risk signatures. Lastly, although experimental studies have confirmed the involvement of certain genes in our risk signature, future research should continue to focus on basic experiments to unravel their interactions and underlying mechanisms in the progression of breast cancer.

## Conclusion

In this study, we developed a novel risk signature based on eight mitochondrial-related genes. Its prognostic accuracy has been validated through various clinical evaluation methods. Additionally, this signature can effectively distinguish between high-risk and low-risk groups among breast cancer patients, thereby suggesting its potential value in clinical applications.

### Supplementary Information

Supplementary Figure 1.

Supplementary Figure 2.

Supplementary Figure 3.

Supplementary Figure 4.

Supplementary Figure 5.

Supplementary Figure 6.

Supplementary Legends.

## Data Availability

The datasets generated during and/or analyzed during the current study are available from the corresponding author on reasonable request.
