# Supplementary figures and images for: Prognosis prediction and risk stratification of breast cancer patients based on a mitochondria-related gene signature
Source: Sci Rep. 2024 Feb 3;14:2859. doi: 10.1038/s41598-024-52981-w (PMC10838276; doi:10.1038/s41598-024-52981-w)

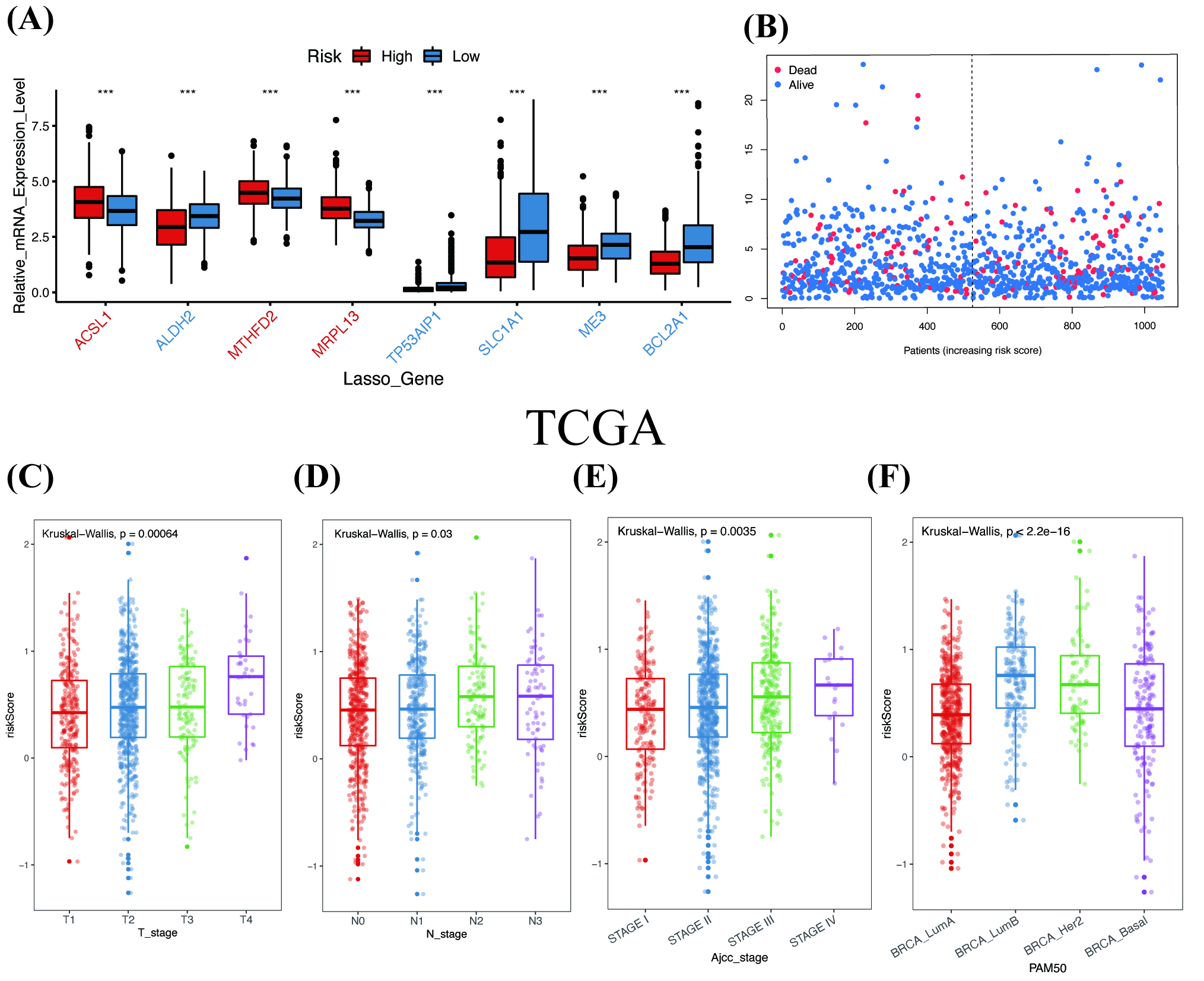

Supplement: Supplementary file 1 — Supplementary Figure 1. [file 41598_2024_52981_MOESM1_ESM.tif]

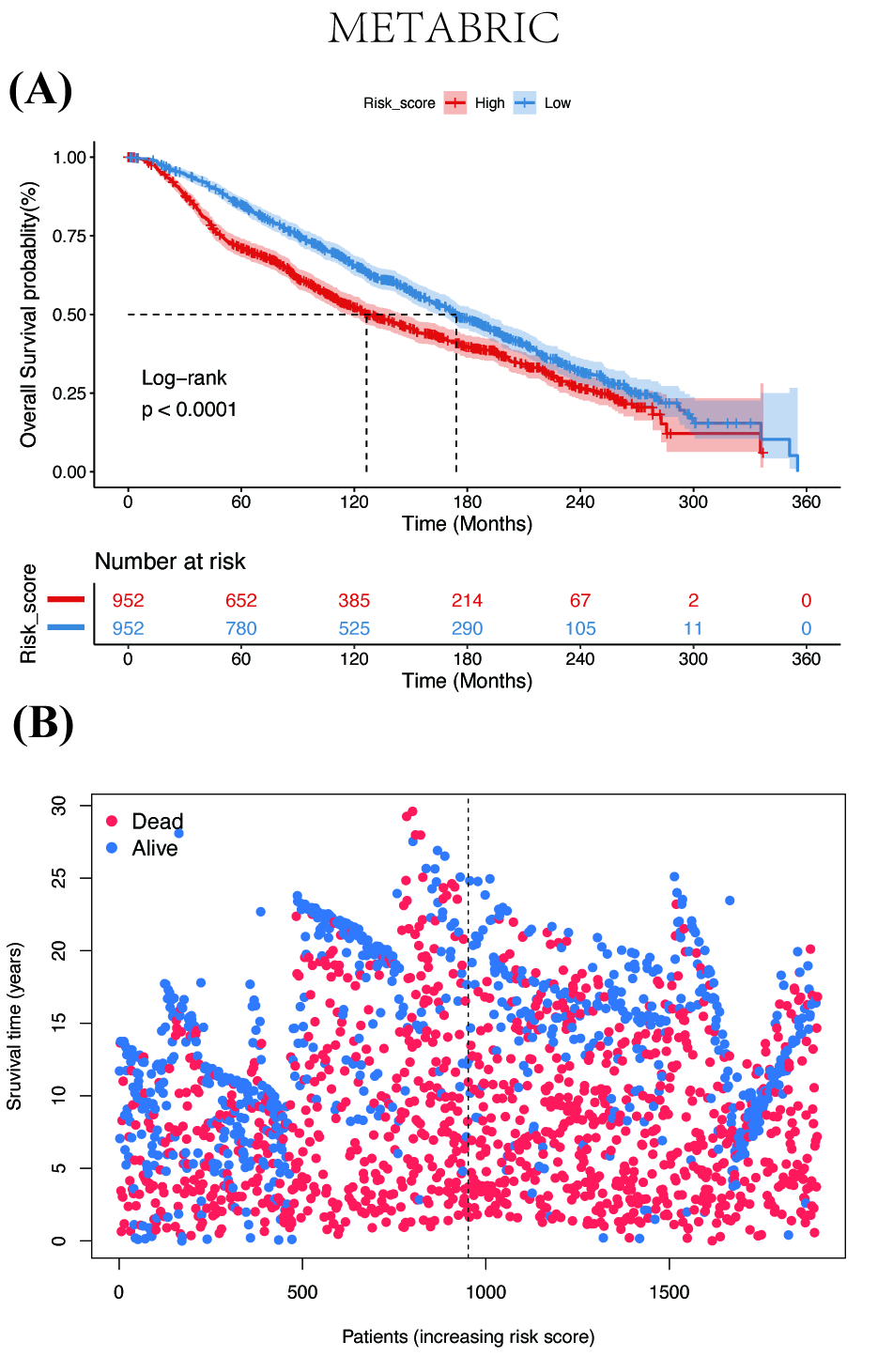

Supplement: Supplementary file 2 — Supplementary Figure 2. [file 41598_2024_52981_MOESM2_ESM.tif]

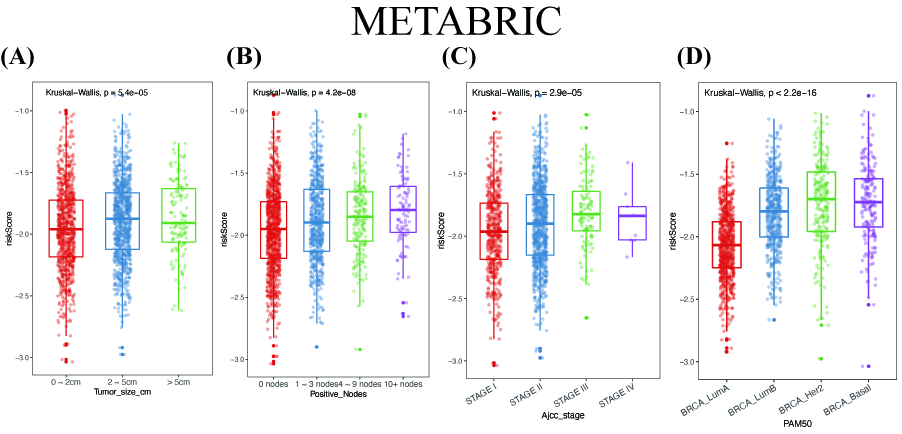

Supplement: Supplementary file 3 — Supplementary Figure 3. [file 41598_2024_52981_MOESM3_ESM.tif]

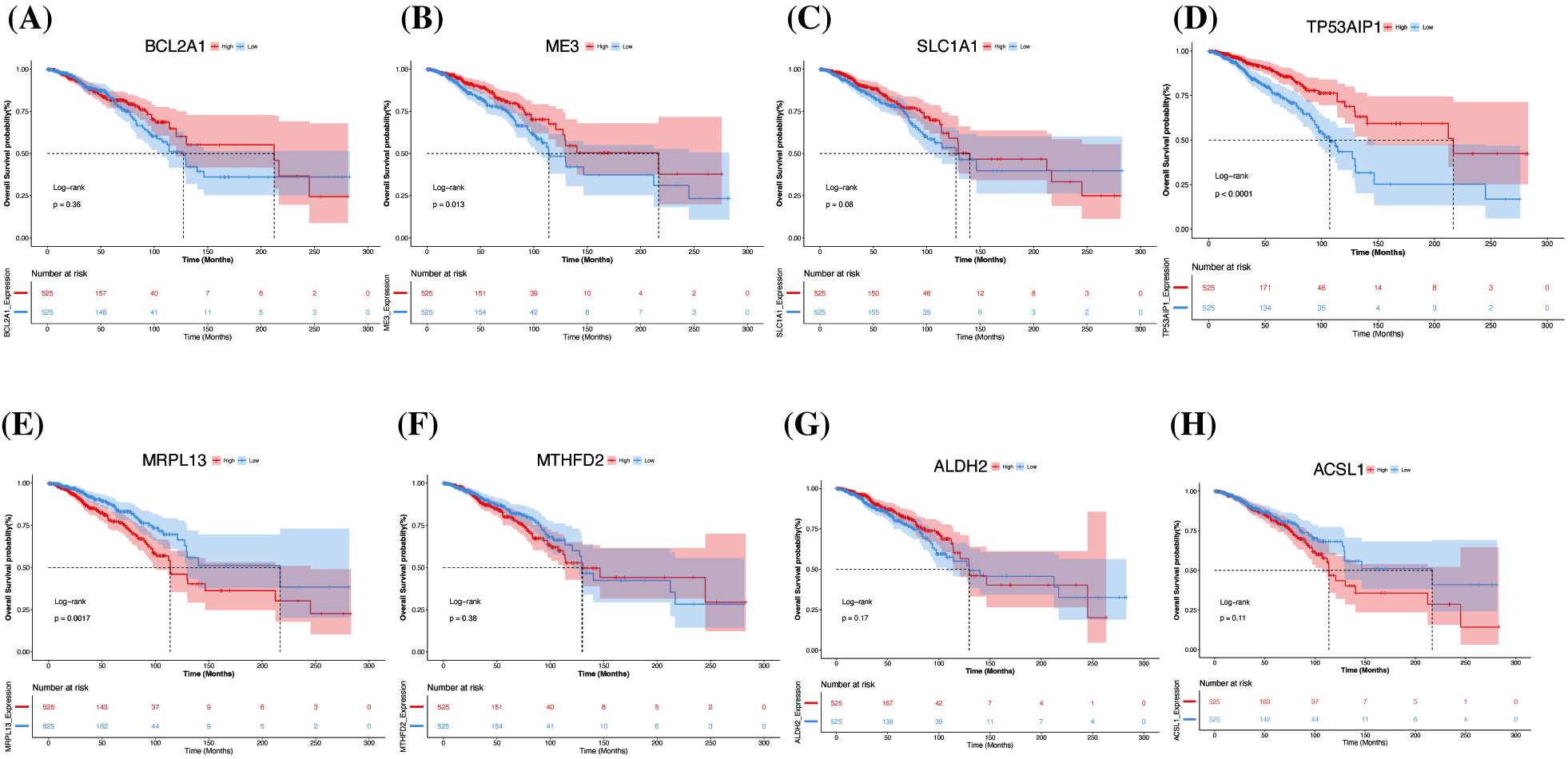

Supplement: Supplementary file 4 — Supplementary Figure 4. [file 41598_2024_52981_MOESM4_ESM.tif]

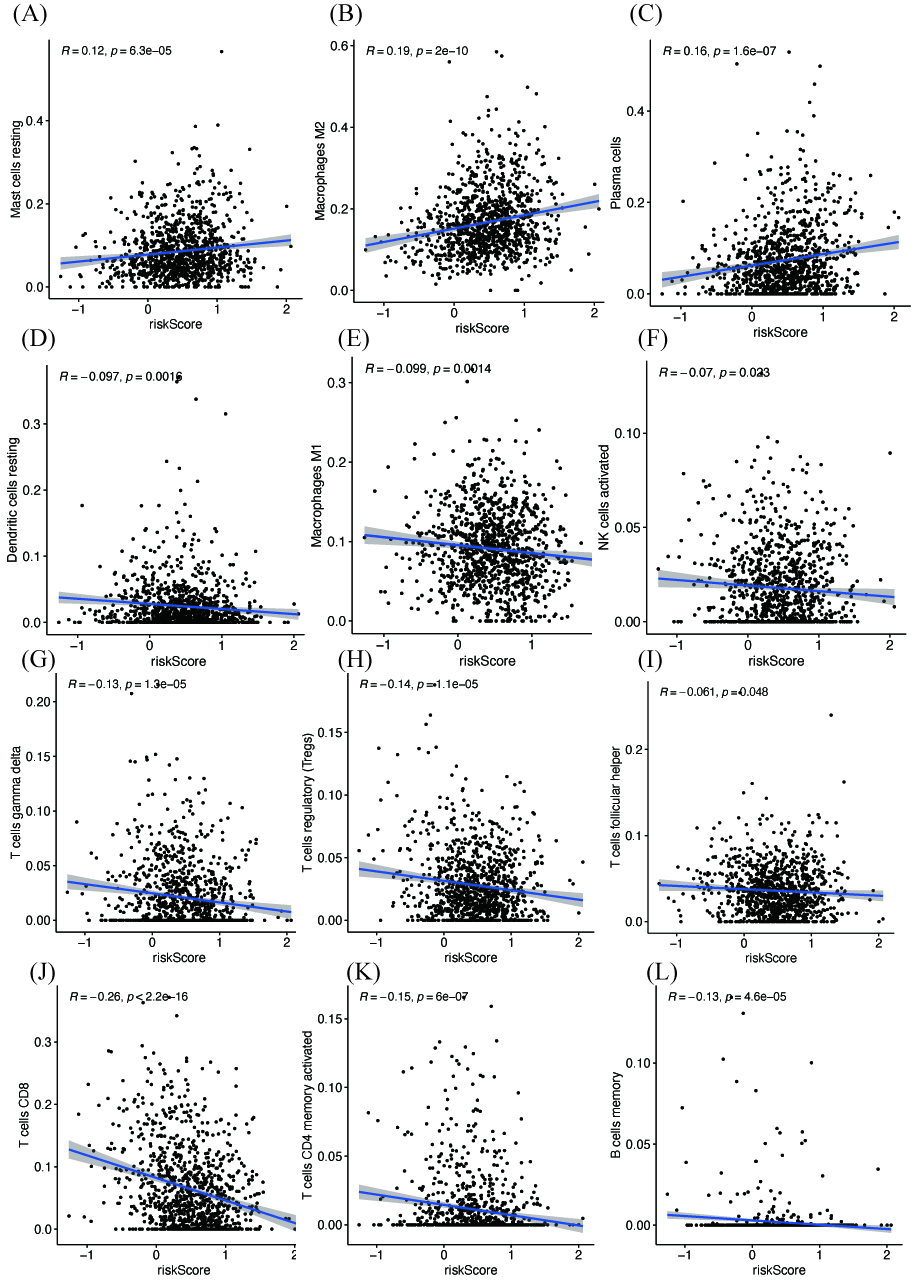

Supplement: Supplementary file 5 — Supplementary Figure 5. [file 41598_2024_52981_MOESM5_ESM.tif]

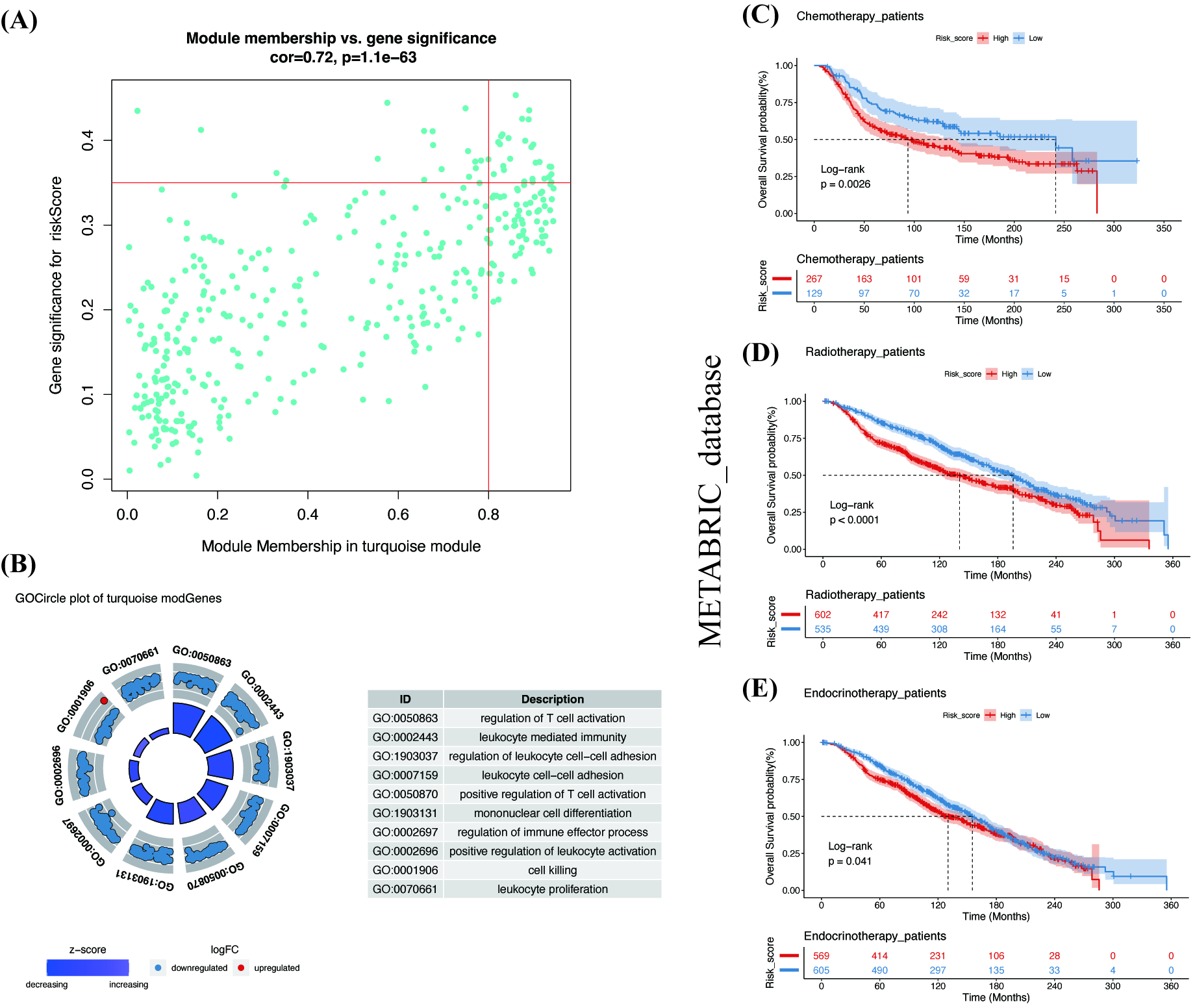

Supplement: Supplementary file 6 — Supplementary Figure 6. [file 41598_2024_52981_MOESM6_ESM.tif]
