# Supplementary material for: Prognosis prediction and risk stratification of breast cancer patients based on a mitochondria-related gene signature
Source: Sci Rep. 2024 Feb 3;14:2859. doi: 10.1038/s41598-024-52981-w (PMC10838276; doi:10.1038/s41598-024-52981-w)
Supplement: Supplementary file 7 — Supplementary Legends. [file 41598_2024_52981_MOESM7_ESM.docx]

***Supplementary figure legends***

**Supplementary Fig 1 Gene expression in the risk signature and correlation between riskScore and clinical features in TCGA cohort.**

(A) The expression levels of the 8 MRGs included in the risk signature between high- and low-risk groups. (B) The survival dot plot for TCGA cohort. Patients were arranged by riskScore on the X-axis (the vertical dashed line represents the median riskScore). (C-F) Boxplot for the distributions of riskScore in different clinical features. (The plot annotations were as follows: * if *P* < 0.05, ** if *P* < 0.01, and *** if *P* < 0.001 and ns if no significantly.)

**Supplementary Fig 2 Survival status of BC patients in MTABRIC cohort**

(A) KM plot for the prognosis analysis between high- and low-risk patients in the METABRIC cohort. (B) The survival dot plot for the METABRIC cohort.

**Supplementary Fig 3 Correlation between the riskScore and clinical features in the METABRIC validation cohort.**

(A-D) Boxplot for the distributions of riskScore in different clinical features.

**Supplementary Fig 4 The prognosis for 8 MRGs in the risk signature**

(A-H) K-M curves for the independent prognostic analysis of 8 MRGs based on their individual median expression level.

**Supplementary Fig 5 CBIERSORT algorithm for tumor-infiltrating immune cells**

(A-C) Scatter plots of cells for the positive correlation between immune cells and riskScore. (D-L) Scatter plots of cells for the negative correlation between immune cells and riskScore. (Only *P* < 0.05 is shown.)

**Supplementary Fig 6 WGCNA and the prognosis for different treatments**

(A) Turquoise scatter plot for the correlation between genes and riskScore examined by Pearson’s test. The x-axis represents module membership, and the y-axis represents gene significance. (B) GO circle plot for the functional enrichment analysis for the turquoise module genes. (C-E) KM plots for the prognostic analysis of patients with chemotherapy, radiotherapy and endocrinotherapy among different risk groups in the METABRIC cohort.
